# A website calculator to benchmark the carbon footprint of haemodialysis

**DOI:** 10.1093/ndt/gfaf263

**Published:** 2026-01-20

**Authors:** Joachim Beige, Susi Knöller, Martin Pachmann, Falk Sommer, Hans Peter Barth, Michael Masanneck, Werner Kleophas, Roman Schaffron, Sylvia Stracke, Kirsten deGroot, Julia Weinmann-Menke, Simone Cosima Boedecker-Lips, Raymond Vanholder

**Affiliations:** Kuratorium for Dialysis and Transplantation, Neu-Isenburg, Germany; Martin Luther University Halle–Wittenberg, Halle–Wittenberg, Germany; Kuratorium for Dialysis and Transplantation, Neu-Isenburg, Germany>; Fresenius Medical Care, Bad Homburg, Germany; Greentec Dialysis, Heidelberg, Germany; Greentec Dialysis, Heidelberg, Germany; Kuratorium for Dialysis and Transplantation, Neu-Isenburg, Germany>; Apollon College of Applied Health Care, Bremen, Germany; DaVita Healthcare, Hamburg, Germany; Heinrich Heine University, Düsseldorf, Germany; DaVita Healthcare, Hamburg, Germany; Kuratorium for Dialysis and Transplantation, Neu-Isenburg, Germany>; Nephology, Internal Medicine A, University Medical Center, Greifswald, Germany; Kuratorium for Dialysis and Transplantation, Neu-Isenburg, Germany>; Sana Hospital, Offenbach, Germany; German Society of Nephrology, Berlin, Germany; Division of Nephrology, Johannes Gutenberg University, Mainz, Germany; Division of Nephrology, Johannes Gutenberg University, Mainz, Germany; European Kidney Health Initiative, Brussels, Belgium; Nephrology Section, Department of Internal Medicine and Pediatrics, University Hospital, Ghent, Belgium

**Keywords:** calculator, carbon footprint, dialysis, greenhouse gas, webtool

## Abstract

**Background:**

Haemodialysis (HD) contributes vastly to greenhouse gas (GHG) emissions. Recognizing this, the German Society for Nephrology initiated a web-based carbon footprint assessment tool to benchmark emissions.

**Methods:**

This study collected data from five pilot HD centres between 2015 and 2023. Emission categories appropriate for HD were defined and included transportation, energy consumption, manufacturing/disposal and other operational factors.

**Results:**

The all-centre, all-period average was 3.72 ± 0.44 tons of carbon dioxide equivalents per patient per year, with manufacturing/disposal, energy consumption and patient transportation as the largest contributions. Over the assessment period, a reduction of 9.1% was achieved, through changes in dialysate flow (−0.16 tons/patient/year), solar power system installation (−0.21 tons/patient/year) and transition to a planetary health–adapted diet (−0.10 tons/patient/year). A best-case scenario with modelled implementation of all ready-to-use measures, including 40% of patients switching to automated peritoneal dialysis and 10% to incremental HD, projected a reduction potential of 38.7% or 1.5 tons/patient/year, substantially less than what is needed to reach net zero.

**Conclusions:**

Using available technology, HD-related GHG emissions were reduced by 9% in the short term. Higher future reductions to meet the targets of a 50% reduction by 2030 and net zero by 2045 might necessitate enhancing prevention and transplantation efforts, technological innovation, support chain adaptations and structural changes like increased use of peritoneal dialysis.

## Graphical Abstract

**Graphical Abstract:**
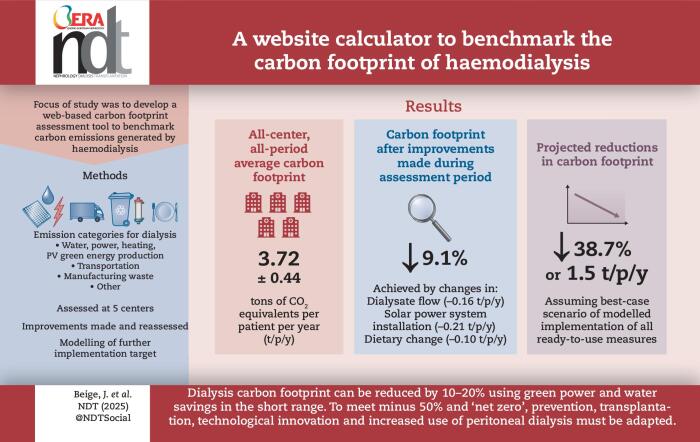

KEY LEARNING POINTS **What was known:**

- Dialysis units contribute significantly to carbon emissions, with each patient generating between 4 to 10 tons of CO_2_ equivalent per year.
- The primary sources of dialysis-related carbon footprint stem from materials, transportation, and energy consumption.

**This study adds:**

- Development of a web-based Carbon Footprint (CFP) calculator, tailored to the specific needs of haemodialysis greenhouse gas (GHG) assessment.
- In a pilot project conducted within German dialysis centers under the National Nephrological Society, baseline GHG emissions ranged from 3.2 to 4.4 tons per patient per year.
- Across all participating centers, GHG emissions decreased by an average of 9%, contingent on the implementation of targeted reduction measures.

**Potential impact:**

- The most effective ready-to-implement strategies for reducing dialysis-related carbon footprint include:
- Optimizing dialysate flow rates, increasing the use of solar-generated green energy, implementing dietary modifications in dialysis snack options
- Pathways to achieving IPCC climate goals can be successfully followed if:
- By 2030, all available immediate-impact measures are universally adopted.
- By 2045, more disruptive innovations are fully integrated across dialysis practices.

## INTRODUCTION

Global climate change imposes significant challenges upon society and urgently necessitates a transition to a more environment-friendly economic model. If humanity does not use the remaining few years of a window of opportunity to limit the global temperature increase to 1.5°C, resulting consequences will progressively worsen across continents [1].

The Intergovernmental Panel on Climate Change (IPCC), a United Nations organization with an objective to provide governments with scientific information that they can use to develop climate policies [2], has defined two necessary targets to be reached in the coming years. By 2030, greenhouse gas (GHG) emissions, including carbon dioxide (CO_2_), should be reduced by 50% compared with 1990 levels. By 2045, at least a 90% reduction (‘net zero’) should be achieved [1].

Healthcare significantly contributes to climate change [3–5] and overall environmental burden, accounting for an estimate of 5–10% of global GHG emissions [6, 7]. Specific features of climate change, such as floods, heatwaves [8] and droughts [9] (partly due to ice shield and glacier loss), and other disasters [10] constitute a specific risk for people with kidney diseases [8, 11]. In turn, dialysis as the last resort to treat chronic kidney disease (CKD) results in high energy and water consumption [12–18], waste production and handling [19, 20] and transportation-related emissions, and is typically repetitive and continued for long periods.

Policy action calls and position statements have been issued by national and supranational societies and working groups. The European Kidney Health Alliance calls on all stakeholders in European nephrology to align with the Green Deal of the European Union [21, 22]. Also, other organizations such as the European Renal Association, the International Society of Nephrology and some national societies and provider groups have issued similar calls [23–27].

In response to these action calls, healthcare providers and kidney and dialysis care communities should upscale their environment-friendly actions to adhere to the IPCC reduction goals [1]. The first step is to have reliable carbon footprint (CFP) data for each kidney replacement therapy (KRT) unit. The dialysis sector needs standardized measurement methods [4], and the currently proposed figures may even underestimate reality. Environmental inventories of kidney and dialysis care should be available at national and/or international levels, enabling comparisons and benchmarking, similar to the current international databases for chemical compositions and toxicological characteristics of industrial materials and products [28, 29].

In 2021, the Kidney and Environment Working Group of the German Society for Nephrology (DGfN) supported the development of a web-linked CFP measuring tool with the intent to open it up to all interested German kidney care professionals. In this publication, the first data from five pilot centres, obtained between 2015 and 2023, are presented. We also describe the methodology and opportunities for benchmarking and reducing haemodialysis (HD) CFP.

## MATERIALS AND METHODS

In 2015, the medical leadership of a dialysis centre in Leipzig took the initiative of calculating its CFP, expressed as GHG emissions, based on consumption values for different factors contributing to the dialysis process. The appropriate conversion for those factors (see below) was calculated by making use of a self-designed Excel spreadsheet (Microsoft, Redmond, WA, USA). Based on this initiative, the DGfN decided in 2020 to standardize this method and make it available to its members for CFP assessment in a larger number of centres. For this purpose, the survey system was made initially accessible via a web portal (www.carbonfootprintdialysis.com) to be used by five centres (Table 1) with the intention of extending this initiative in the future to all centres of the DGfN. Centre operators were able to enter their data using the online form. When real-time data checks revealed incomplete or incorrect datasets, additional checklists and an Excel template were provided, enabling supported online data entry or manual transfer of tabulated data. To avoid double reporting, online data entry was disabled if centres preferred manual transfer. After closing the data entry period, no further requests for online transfer from external centres were retrieved via the website (https://greentecdialysis.com/de/co2-rechner/).

**Table 1:** Characteristics of participating centers for the year 2023.

	Emden (#1)	Bremen (#2)	Offenbach (#3)	Greifswald (#4)	Leipzig (#5)
Geographical location in Germany	North Coast	North	Center West	Baltic Coast	Center East
Electricity production/y (kWh)	0	0	0	0	60.600
Electricity consumption/y (kWh)	234 426	166 559	162 679	125 923	123 998
Proportion of HDF sessions (%)	21	1.7	0	8.3	2.5
Machine types	FMC 5008,	Nikkiso DBB07,	Nikkiso Exa	FMC 5008,	Nikkiso DBB07,
	5008S	Nikkiso Exa		5008S	Nikkiso Exa
Room heating consumption/y (kWh)	329 100	79 465	82 000	50 750	73 230
Room heating energy source	Oil	Gas	Gas	Gas	local remote
R/O water consumption/y and Δ 2021 (m^3^)	5 191; 482	2 263; 517	3 311; 319	3 214; 350	4976; 648
HD patients (n)	82	80	84	78	147
HD sessions/y (n)	14 300	12 363	12 716	12 504	24 052
Number of attending staff / w (n)	116	84	90	57	76
Session duration (min)	255 ± 43	275 ± 64	256 ± 19	252 ± 29	269 ± 59
Center surface area (m^2^)	920	1 420	1 499	725	2 605

w = week, y = year. Staff attendances were counted for each attendance day per week. Locale remote heating means a local combined heating and electricity power plant serving the hospital and adjacent area.

### Facility-based emission impact

Emissions were calculated based on the data entered for the HD activities or the properties of each participating centre and adapted to emission types according to ‘sectors or scopes’, which are standard classes in sustainability literature [30–32], including:

- transportation emissions (for patients and staff—proportional to fossil fuel and electric energy source; scope 2 emission types);
- emissions for manufacturing of consumables (except their transport) following a weight-based method [15] and, separately, emissions for handling the derived waste, with the calculation based on waste weight (scope 2; see Supplemental Data for details);
- emissions attributed to consumption of power, room heating and water, including preparation of dialysis water by the reverse osmosis (RO) unit, composed of RO manufacturing CFP and RO power CFP (scope 2). Consumption of solar-produced power was calculated with a reduced CO_2_ conversion factor (see Supplemental Data), based on the readouts at the corresponding meters in the centres; and
- emissions attributable to other reasons, like those related to laundry, cleaning, nutrition and informatics (scope 2).

Variables to be provided by the centres at/before an annual cut-off date and relevant to the emission categories mentioned above are given in Table 2. A detailed description of the methods used for emissions assessment can be found in the Supplemental Data. Next to facility-based analyses, separate analyses were performed to allow the assessment of the effect of some specific measures on the dialysis CFP.

**Table 2:** Variable input in website calculator for the year under consideration.

Emission category	Variables to be provided and used for calculation
Transport	Patients (n), proportion of electrical cars for patient transport (%), average travel distance of staff and patients to center (km), proportion of bicycle, public and personal electric transport for staff travel (%)
Consumables and waste	Patients (n), HD sessions per year (n), estimated waste weight (kg)
Electricity, heating, raw water delivery	Patients (n), electrical energy consumption (kWh), electricity production (kWh),water consumption (m^3^), room heating consumption (kWh)
Other (laundry, cleaning, nutrition and IT)	Patients (n), HD sessions per year (n), surface area for center use (m^2^), IT workstations and printers (n), snack rolls (n) and type of diet, frequency of bed cover laundry

### Impact of specific measures

#### Dialysate flow (***Q***_d_)

The basis for *Q*_d_ individualisation was a provider-wide consensus and recommendation to use individualized flows. A preset machine *Q*_d_ was reduced to 350 ml/min. Individual dialysis prescription was performed by attending physicians after informing patients and considering parameters like target dialysis dose, dialyser blood flow and detoxification needs, residual urinary volume, body weight, clinical condition and age. The effects of *Q*_d_ reductions, including impact on RO, were estimated by measuring facility-based power and raw water consumptions at the corresponding metres between full years before and after flow changes. To assess the impact of *Q*_d_ reduction on solute concentration, serum phosphate (PO_4_), potassium (K) and bicarbonate (HCO_3_) levels were measured in people in whom *Q*_d_ was reduced at first-in-week-sessions 4 weeks before and after change in centre 5.

#### Installing solar panels

The impact of solar power panel instalment was tested by comparing electricity consumption in one centre, where solar panels of 88 kW peak power were installed in March 2016, with an extension of the solar panel park in late 2023 to 112 kW peak power. For this centre, a comparison in emissions was made between 2015 [no photovoltaic (PV) system] and the average of 2017 and the period from 2020 to 2023. Of note, all these data were obtained before *Q*_d_ was modified.

#### Patient transportation modifications

Differences in GHG emissions were calculated between individual and group patient transportation in one centre by comparing data from the years before the COVID-19 pandemic, when group transportation for two to three patients in one vehicle was used, with the pandemic years when individual transportation was the rule.

#### Diet modifications

The effect of diet type was modelled by calculating GHG emissions based on predefined emission scales for diet components of the intradialytic meals provided in one centre, where the diet type was changed, after informing and educating patients by the attending physician (S.K.) from 2020 to 2021, from a conventional diet with meat cold cuts or cheese, bread and rolls to a diet based on fruits, vegetables and curd [33].

#### Maximum potential benefit

Finally, we also made a calculation of the maximum potential reduction in CFP if all participating centres would maximally exploit possible solutions for each category and a switch to automated peritoneal dialysis (APD) with a lower CFP than HD [12] in 40% of all patients plus including an incremental HD initiation policy [34] (starting 50% of patients twice weekly for 6 months and continuing 10% on twice weekly for 2 years).

### Statistical analysis

Facility-based and per emission category CFP were first computed per centre and subsequently normalized for the patient number and expressed in tons/patient/year. No data were collected on home dialysis. Descriptive statistics are represented as mean ± standard deviation after control for normal distribution. To compare CFP data per patient from year to year and between centres, paired and unpaired *t*-tests and analysis of variance were used where appropriate.

## RESULTS

### Facility-based emission impact

**Figure 1:**
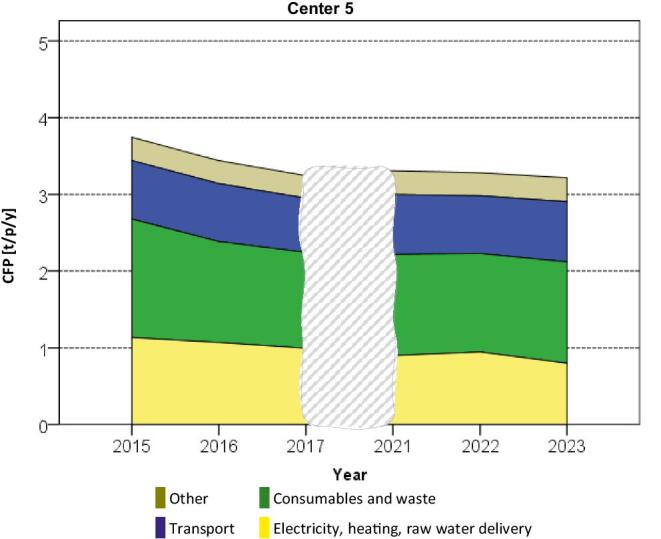
Time course of carbon footprint (per sector and facility-based) per patient in centre 5 from 2015 to 2017 and 2021 to 2023 (yellow: electricity, room heating, raw water delivery; blue: transportation; green: material and waste; ochre: other (laundry, cleaning, nutrition, information technology).

Centre 5 started data collection in 2015 and reduced the CFP from 3.74 to 3.44 in 2016 (−8%) and 3.24 in 2017 (−13%). After an interruption, data registration was restarted in 2021, at the moment of enrolment of four additional centres, with relatively stable values: 3.31 in 2021, 3.28 in 2022 and 3.22 in 2023 [all in tons/patient/year; overall change from 2015 to 2023 −0.52 tons/patient/year (−14%)] (Fig. 1).

The average total dialysis CFP from the five centres under study in the period 2021–2023 covering 15 centre years is shown in Table 3, that contains averages per category for 2021 and 2023, the changes between 2021 and 2023 for the five centres together and CFP results per category and centre for 2023.

**Table 3:** Overall CFP and CFP per category per patient in 2021 and 2023.

	Period		Changes			Single center values 2023					
Category CFP(t/p/y)	2021	2023	t/p/y	%	p^+^	1	2	3	4	5	p*
Transportation	0.77 ± 0.27	0.76 ± 0.28	−0.005 ± 0.009	−0.74 ± 1.40	0.46	0.55	0.76	0.50	1.21	0.79	<0.0001
Consumables and waste	1.45 ± 0.13	1.40 ± 0.11	−0.033 ± 0.102	−3.31 ± 6.58	0.36	1.4	1.42	1.55	1.31	1.32	0.14
Electricity, heating, raw water delivery	1.48 ± 0.56	1.17 ± 0.44	−0.303 ± 0.158	−20.5 ± 7.51	0.01	1.92	1.03	1.18	0.94	0.80	<0.0001
Other (laundry, cleaning, nutrition and IT)	0.22 ± 0.082	0.22 ± 0.081	−0.003 ± 0.008	−1.36 ± 2.89	0.49	0.28	0.21	0.18	0.11	0.31	<0.0001
**Overall**	3.91 ± 0.60	3.56 ± 0.35	−0.356 ± 0.257	−9.10 ± 5.01	0.04	4.15	3.43	3.41	3.57	3.22	<0.0001

*ANOVA, ^+^paired t-test.

Overall, CFP was 3.91 ± 0.60 tons/patient/year in 2021, 3.76 ± 0.49 tons/patient/year in 2022 (−4%) and 3.56 ± 0.35 tons/patient/year in 2023 ( −9%; *P* = .04). There was also a significant decrease over time for the category comprising electricity, raw water delivery and heating.

Between centres, there were significant differences concerning the facility-based GHG emissions and category emissions for electricity, heating and raw water delivery, transportation and the item categorized as ‘other’. More detailed results comprising all retrieved data are presented in the Supplemental Table S1.

**Figure 2:**
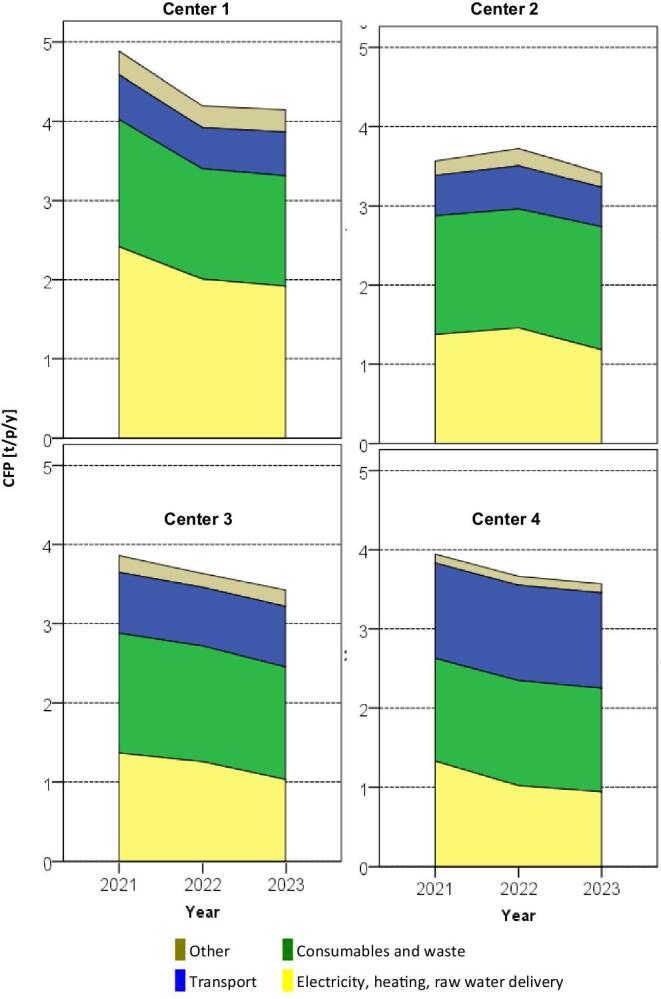
Time course of carbon footprint (per sector and facility-based) per patient in the remaining four centres between 2021 and 2023 (same colour coding as in Fig. 1).

Figure 2 shows the evolution of CFP for each of the four centres that participated in the registration only between 2021 and 2023. Similar to the centre starting in 2015, the main contributors to GHG emissions were material production, waste handling and energy consumption. Overall, a decreasing trend was observed that was related to a decrease in electricity consumption and lower volumes of raw water delivery, as well as heating-related emissions.

According to these data, the additional per-person (treatment-related) CFP of people in need of in-centre HD adds ≈40% (≈3.6 tons/patient/year) to the general German GHG emissions per person, which ranged from 9.15 tons/patient/year in 2021 to 8.02 tons/patient/year in 2023 [31, 35, 36].

### Impact of specific interventions

#### Dialysate flow reduction

Centres reduced *Q*_d_ in 50–80% of their patients by applying individualized adaptations to HD prescriptions (personal communication). Aggregated facility-based CFP values for electricity and raw water delivery changed from 1.02 ± 0.31 to 0.86 ± 0.26 tons/patient/year (*P* = .07). Water consumption of the RO decreased by an average of 14% (Table 3). While four of five participating centres could reduce electricity plus raw water CFP significantly, this was not the case in centre 2, where, due to unrelated confounders (see discussion), net electricity consumption increased after the conversion.

**Figure 3:**
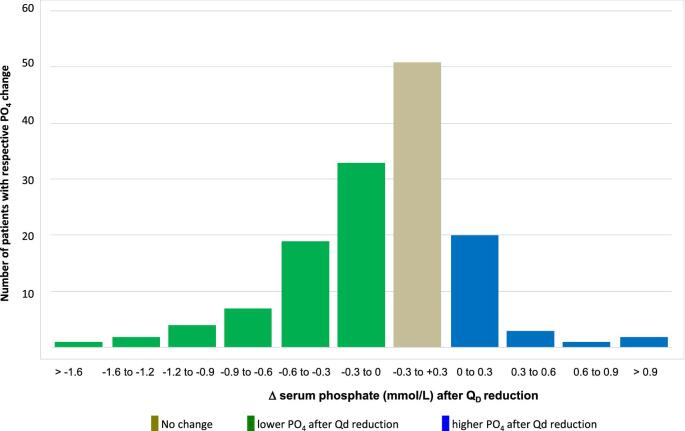
Distribution of patients according to changes of their serum phosphate levels after conversion from 500 to 350 ml/min dialysate flow (green: patients with lower PO_4_ after conversion; grey: no change; blue: patients with higher levels after conversion.

In centre 5, first-day-in-week, pre-HD serum phosphate, potassium and bicarbonate values in patients in whom *Q*_d_ was reduced were 1.80 ± 0.50, 5.29 ± 0.57 and 20.3 ± 8.48 mmol/l 4 weeks before reduction versus 1.77 ± 0.51, 5.70 ± 1.01 and 19.9 ± 7.95 mmol/l 4 weeks thereafter [changes in mmol/l, −0.04 ± 0.46 (*P* = .3); + 0.41 ± 0.98 (*P* = .54); −0.40 ± 0.53 (*P* = 0.32) (Fig. 3)].

#### Installing solar panels

In centre 5, the CFP for electricity was reduced from 0.84  tons/patient/year in 2015 (without own electricity production) to 0.63 ± 0.03 tons/patient/year in 2017 and in 2021–2023, when it had an operational PV system (−22 ± 3.57%).

#### Transportation changes

In centre 5, CFP per patient for transportation was smaller (0.61 ± 0.01 tons/patient/year) when group transportation was in use before the pandemic, compared with 0.64 ± 0.01 during the pandemic years with separate transportation for everyone (*P* = .03, paired *t*-test).

#### Diet modifications

Transition to a healthy diet was associated with a change in estimated nutrition-related CFP from 0.19 to 0.09  tons/patient/year, which is a decrease of 53% in the nutrition category and 3% facility-based [33].

### Maximum potential benefit

Finally, we also used the website calculator to estimate the optimal GHG reduction if measures were applied as efficiently as reasonably possible (Table 4). This best-case scenario contains either measured reductions or modelled data based on published data. Applying optimally all ready-to-use measures, including 40% of patients switching to APD and 10% to incremental HD, projected a reduction potential of 38.7% or 1.5  tons/patient/year.

**Table 4:** Optimal reduction possibilities based on the current data and best practice solutions.

	CFP (t/p/y or %)				
	Prevalent Emissions	Measured or		Absolute	% Reduction of
Category	per patient/year	calculated (*) Action	Optimal Emissions	Reduction	total CFP
Pat. transport	0.64 ± 0.007	100% electric transport*	0.44	0.2	5.2
Staff transport	0.15 ± 0.03	50% non fossile commute*	0.07	0.07	1.8
Electricity, heating, raw water delivery	1.02 ± 0.31	Dial. temp. reduct.	1.01	0.011	0.28
	0.84	PV	0.63 ± 0.03	0.21	5.4
	1.02 ± 0.31	Qd reduction	0.86 ± 0.26	0.16 ± 0.15	4.2
Other	0.19	Planetary diet	0.09	0.10	2.6
**Subtotal**	**3.86**		**3.10**	**0.75**	**19.5**
Mode change	–	40% of pts. on APD**	–	0.66**	17.1
Mode change	–	Incremental start (50%), 2yr (10%) ***	–	0.062	1.6
**Total**	**3.86**		**2.38**	**1.47**	**38.7**

Each item is displayed with the optimal theoretical response, or if data from several centers were available, with the result of the best performing center. Items with * derived from literature or calculations. **The effect of promoting PD (target 40%) was calculated by annual emissions for in-center HD (3.86) minus emission for automated (APD (2.2) = 1.66 multiplied by 40%. The starting point to calculate reductions by APD was the average of the 5 pilot centers in 2021. *** The starting point of incremental HD was calculated by assumed proportions of 50% starting HD 2x/weeks over 6 months and 10% remaining 2x/week with an estimated survival time of 2 years.

## DISCUSSION

The presented data retrieval platform enables a standardized web-based survey of the CFP of HD units, offering an opportunity for a full-scale national survey in the near future.

The magnitude of dialysis CFP in the German centres included in this study was similar to that found in studies from the UK [13] and Morocco [14], with a per-patient CFP of 3.8 and 5.1 tons/patient/year in 2010 and 2020, respectively, but lower than in the USA (9.2  tons/patient/year) [15] and Australia (10.2  tons/patient/year) [16]. However, the scope of categories and methods of data collection differed between studies, making direct comparisons difficult. The UK study used a component analysis approach, while our study used facility-based consumptions, travel kilometres, material weight and waste weight normalized to the number of patients. With 2.6 tons/patient/year for electricity and 2.8 tons/patient/year for transportation in the US study, these categories seem to be responsible for the higher emissions compared with our European data and presumably, as in Morocco, more electrical power is needed for climatization. Sehgal *et al.* [15], in their study based on dialysis units in Ohio, described discrepancies in GHG emissions among centres, which according to our findings is also appropriate for Germany. The role of building insulation was underscored by the higher GHG emissions related to heating in centre 1 of this study, which is housed in interconnected circular satellite buildings with large floor-to-ceiling windows and glass domes. This building, although of architectural interest, offers no opportunity for improvement of thermal insulation. This creates a dilemma between the difficulties of matching architecture with sustainability on the one hand and the environmental burden of leaving an existing building potentially unused and replacing it with a new additional structure on the other. Irrespective of this, in all studies the most important dialysis-related CFP categories were electrical power consumption, patient and personnel transportation and emissions of manufacturing and waste handling of dialysis materials.

Methodological differences among studies produce a debate on which categories should be included in dialysis CFP calculations and whether the dialysis CFP should strictly adhere to the ‘scopes’ known from the non-medical sustainability literature [30, 32]. GHG emissions for electricity consumption (including internal dialysis water preparation by the power-consuming RO) and external water delivery (from a water plant to the HD unit) were merged into one category.

In our study, consumable production and waste handling, both measured by weight, contributed to 1.3–1.6 tons/patient/year of GHG emissions, or 40% of total facility-related CFP. In an Australian study on PD, it appeared that variations in transportation distances of materials had a significant impact on differences in emission [12].

Given the challenges of achieving harmonized CFP assessment for dialysis across countries and continents, we suggest standardized assessment tools to be disseminated within specialist organizations and among providers and, importantly, allowing comparisons over time. Our tool integrated time-course data, allowing us to analyse sustainability measures taken by individual centres.

Some of the most important CFP reductions resulted from installing solar power (in one centre) and reducing dialysate flow (in all centres). Each measure allowed a reduction in the range of 0.15–0.20 tons/patient/year and both were major contributors to the overall decrease of total dialysis CFP in our hands by ≈10%.

Most outpatient dialysis centres operate during daytime, and their roofs are often very suited to install PV systems. A centre with ≈150 patients and a 400 m^2^ roof can expect a peak power of 100 kW on sunny days, covering the energy needs of 30–40 treatment positions and enabling electrical self-sufficiency between April and October. To assess the environmental impact of green electricity, we included life cycle CFP of solar panels in the conversion factor [18, 28].

A success attributable to a decrease in dialysate consumption was observed in four of five centres, with one centre showing no change, very likely related to construction works consuming electricity that could not be separated from medical consumption.

In the largest centre (centre 5), phosphate, potassium and bicarbonate levels did not show relevant changes after dialysate flow reduction. Canaud *et al.* [38] showed that haemodiafiltration (HDF) offers a possibility to reduce dialysate flow while maintaining adequacy, due to the added value of convection. However, in our study the HDF is unlikely to have played a role, as only 2.5% of patients were treated by HDF.

In line with our findings of unaltered phosphorus, bicarbonate and potassium despite dialysate flow reduction, two single-centre studies that investigated the effect of reduced dialysate flow from 500 to 300 ml/min on urea removal by assessing *Kt*/*V* showed no changes [39, 40]. In contrast, a meta-analysis of *Q*_d_ increase from 500 to 800 ml/min reported an increase of *Kt*/*V* by 0.08 but without specifying relative (%) changes [41]. Hence, careful clinical endpoint studies including *Kt*/*V* and relevant protein-bound molecules and small polypeptides (‘middle molecules’) are needed to define the optimal amount of dialysate flow, balancing environmental friendliness, dialysis adequacy and clinical appropriateness. To comprehensively address these issues we support the need of individual dialysis prescriptions avoiding water and energy waste by minimizing redundant dialysate flows and defining in advance key performance indicators [26]. Such balance between environmental measures and dialysis adequacy is an issue that deserves further study. There is also a need for educational programs addressing patients to make clear that environmental issues should not be detrimental to quality of life and outcomes of those treated. At our current level of knowledge there are few if any data of a causative association between decreases in dialysate flow rates and negative outcomes or decreased adequacy parameters. Yet the patient should be informed about flow changes as for any other change in their dialysis protocol, with information on the reason and the possibility to refuse or step out, as was the case in the present initiative.

Switching to a planetary-health diet was highly efficient to reduce CFP related to nutrition by ≈50%, highlighting its combined benefit for health and environment and its suitability for a multiplied effect when education for home nutrition is provided as well.

Emissions related to the transportation of patients and staff accounted for 14–32% of the total dialysis CFP. After the suspension of customary group transportation due to the pandemic, only a small increase of transportation-related CFP occurred. However, pre-pandemic group transportation occurred without systematically tracking travel kilometres, but considering unit organization (time schedules) and costs rather than environmental impact. Better planning of group travel with a focus on minimizing tour lengths could result in more significant fossil fuel reductions and must be strived for. Another option could be using cars powered by electricity charged preferably from solar panels. Assuming a daily solar electricity harvest from a large panel array of 600 kWh daily, a GHG-free distance reach of 2000–3000 km can be achieved, enough for transportation of 40–70 patients during 1 week, based on an average transportation distance of 13 km. Further GHG emissions savings could be accomplished by dialysis personnel walking, cycling or using public or non-fossil fuel transportation to travel to the unit.

This study has some limitations. Concerning platform performance, data quality checks during the retrieval period showed a need for further development of a user-guided and plausibility-controlled data entry process into the system. In addition, emissions for transport of consumables, building construction and manufacturing and transport of medications could not be calculated, because reliable data were lacking.

Consumables (dialysers, tubing systems, concentrate cartridges, fluid bags, needles and syringes) were considered based on their weight [15]. Future projects applying non-incineration and material recycling [42] would gain informative value if they are based on measured emissions rather than on indirect values. Consequently, there is a need for the pharmaceutical and dialysis industries to develop consumption-based CFP assessment principles, based on production and delivery processes, and to make the data available to the public. Clarifications on their optimization measures would help consumers compare and make weighted choices promoting environment-friendly practices, as reportedly up to 70% of healthcare-related GHG emissions come from the supply chain, including production, transportation and waste handling [7, 17]. Even if material transport is not included in our analysis, using central dialysate concentrate mixing devices is another option to reduce the transport resources needed for delivery of heavy dialysate concentrate containers [43, 44].

To inform policymakers, we demonstrated that the dialysis CFP of pioneering centres could be reduced by ≈9%, and in a perfect world by ≥20%, with measures available today, particularly renewable solar energy and reductions in dialysate flow.

Further reductions may be more difficult to achieve without proceeding to alternative more environment-friendly treatment modes and/or technologies. HD procedures at home may result in environmental savings, primarily related to less transportation and room climatization unless dialysis time and frequency are increased without a proportional decrease in dialysate flow. The greater potential of home haemodialysis (HHD) to apply alternative time frames creates a conflict between treatment efficacy and ecological impact. Connor *et al.* [17] calculated a CFP of 3.4 tons/patient/year for HHD at 3 × 4 h/week compared with 3.8 tons/patient/year in-centre. However, this benefit was lost with increased dialysis frequency or length with unmodified dialysate flows, with at the upper extreme of a CFP of 7.2  tons/patient/year at 6 × 7 h/week.

PD, although not environmentally inert, may be even more favourable than HHD, generating an estimated CFP of 1.4 tons/patient/year for continuous ambulatory peritoneal dialysis and 2.2 tons/patient/year for APD [12, 13, 18], lower than what was found for HD procedures in this study. In line with personalized KRT regimes, incremental HD schemes with reduced weekly frequencies, if medically appropriate, provide reductions of energy and water consumption [34, 45].

By applying all available possibilities, including a major switch to PD and including incremental schemes, dialysis centres may come close to achieving the IPCC target of 50% lower GHG emissions in 2030. But additionally, to reach net zero by 2045, alternative methods will be required, such as application of emissions-reducing materials, material recycling and dialysate reprocessing [46, 47].

Finally, the smallest CFP related to KRT for kidney failure would be reached if a minimum of dialysis is needed by optimal prevention and transplantation. Appropriate screening, along with administering novel drugs reducing the progression of CKD [48–50] could have a significant effect on GHG emissions by avoiding KRT. Theoretically, each avoided dialysis year saves ≈4 tons of GHG per patient and during a 20-year course, a 40% anti-progressive effect (dialysis postponed by 8 years) would result in up to 32 tons overall per patient. A real-data secondary analysis of the placebo-controlled CREDENCE study investigating the potential of canagliflozin on outcomes found a 20–25% GHG reduction (39 kg/patient/year) during 2.6 years of follow-up in patients with type 2 diabetes not yet on KRT [51]. A comparable amount can probably be saved by each additional transplantation, although data are currently only available for liver transplantation, for which it was shown that the surgical procedure plus follow-up did not cause significant GHG emissions when compared with long-lasting supportive treatment without transplantation [52]. A 10% increase in the German kidney transplantation rate would thereby avoid ≈800 tons of CO_2_/year.

In conclusion, we showed that implementing renewable energy at the facility level and optimizing dialysate flow can reduce the CFP of HD by ≈10%. However, taking only in-centre measures may not suffice to reach a 50% reduction by 2030, which probably is possible only by promoting a switch to resource-saving strategies such as PD and additional optimization of transportation of people remaining in-centre. New technologies like dialysate regeneration and material recycling may further decrease emissions to target net zero by 2045.

## Supplementary Material

gfaf263_Supplemental_Files

## Data Availability

The data underlying this article are available in the article itself.
