# Supplementary material for: A website calculator to benchmark the carbon footprint of haemodialysis
Source: Nephrol Dial Transplant. 2026 Jan 20;41(7):1294–303. doi: 10.1093/ndt/gfaf263 (PMC13403270; doi:10.1093/ndt/gfaf263)
Supplement: gfaf263_Supplemental_Files [file gfaf263_Supplemental_Files.zip › green_dialysis_r2suppl.docx]

**A Website Calculator to Benchmark the Carbon Footprint of Haemodialysis**
*Joachim Beige^1,2^, Susi Knöller^1^, Martin Pachmann^3^_,_ Falk Sommer^4^, Hans Peter Barth^4^, Michael Masanneck^1,^* ^5^*, Werner Kleophas^6,7^, Roman Schaffron^6^, Sylvia Stracke^1,8^, Kirsten deGroot,^1,9^, Julia Weinmann-Menke^10,11^, Simone Cosima Boedecker-Lips^11^, Raymond Vanholder^,12,13^*

*^1^*Kuratorium for Dialysis and Transplantation, Neu-Isenburg
*^2^* Martin-Luther- University Halle/Wittenberg
*^3^*Fresenius Medical Care, Bad Homburg
*^4^*Greentec Dialysis, Heidelberg
^5^Apollon College of Applied Health Care, Bremen
*^6^*DaVita Healthcare, Hamburg
*^7^*Heinrich-Heine-University, Düsseldorf, Germany
*^8^*Nephology, Internal Medicine A, University Medical Center, Greifswald
*^9^*Sana Hospital, Offenbach
*^10^*German Society of Nephrology, Berlin
^11^Div. of Nephrology, Johannes-Gutenberg-University, Mainz
[all in Germany]
*^12^*European Kidney Health Initiative, Brussels, Belgium
^13^Nephrology Section, Department of Internal Medicine and Pediatrics, University Hospital, Ghent, Belgium

Correspondence address
Prof. Dr Joachim Beige
Kuratorium for Dialysis and Transplantation
Delitzscher Straße 141, 04129 Leipzig - GERMANY
Joachim Beige@kfh.de

**Supplemental text**

**Methods used for emission assessment**

Key characteristics are constructed to allow open access in Germany for centers on an annual basis, with a user-friendly design, necessitating only 60 minutes for data input per year, CFP determination according to standardized categories and analysis of changes over time and comparison with the available literature data.

The tool was created using Zenforms (Axonic, Karlsruhe, Germany, [1]) and embedded into the website and corresponding Excel sheet. After checking the incoming comma-separated-value (CSV) data for plausibility by the website operator (HPB), analysis was performed using specifically programmed Phyton (Delaware, USA) elements.

The GHG emissions relevant for the chosen dialysis categories were calculated using CO2 equivalence conversion factors from the databases Ecoinvent, GEMIS, IFU, Probas, Öko-Institut and LiveLCA [2–6] attributable to all items included in table 2 and updated to the appropriate time period. Calculation was performed according to the French Agence de la Transition Ecologique (ADEME [7, 8]) directive, the World Resources Institute (WRI) and the World Business Council for Sustainable Development (WBCSD [9]). As an example in 2022, 2023 and 2024 in Germany, one kWh electricity consumption had a defined GHG emission factor of 0.48, 0.43 or 0.28 tons of CO2 equivalent, respectively. Such conversion factors and similar ones for kilometers of travel, kilograms of waste, m^3^ of raw water supply (excluding RO energy need, which was included by meters in the centers) etc. change over time following the proportion of renewable energy and production conditions applicable during a particular year in the specific country.

For the corporate carbon footprint, the system organisational and operational boundaries must be clearly defined and presented in a plausible and comprehensible manner [10].

The organisational system boundaries describe the organisational unit and the period to which the carbon footprint relates. The operational system boundaries describe the emission sources that are taken into account within the organisational boundaries.

To distinguish between different emission sources, the GHG Protocol distinguishes between three categories (‘scopes’, [11]). The accounting period covers greenhouse gas emissions for one year, beginning on 1 January and ending on 31 December.

The relevant categories for the healthcare sector are taken into account in accordance with the ADEME guideline [8].

**Scope 1 emissions:**

Direct emissions from own combustion (combustion of fossil fuels in boilers, etc.).

**Scope 2 emissions:**

Indirect emissions from the purchase of grid-bound energy (electricity including specified lower but not ZERO factors for PV (“green) energy, heating, cooling)

**Scope 3 emissions:**

Other emissions from processes caused directly or indirectly by the producer of purchased goods and services, e.g consumables, food, raw water procurement and infrastructure for preparation of dialysis water excluding the RO energy needed for it´s generation (as specified in scope 2). Electricity consumption of the RO device was included in the global electricity consumption of the unit. The following scope 3 emissions are not included as they fall outside the system boundaries we have defined: a) Furniture and other equipment, b) medical devices, c) medicines, d) buildings.

Emissions of manufacturing and waste handling (separated) of the dialysis consumables were estimated based on the dry (before-use) weight or the waste weight after treatment, resp. [12].

We used a flat rate based on the average weight of waste per session. The average value was determined based on the total annual weight of residual waste from 12 centres as measured by the waste disposal company and normalised to the number of sessions. Clinical (contaminated) waste for incineration was recorded separately from waste for recycling (mainly packaging), and appropriate conversion factors were applied.

Travel distances and modes for patients and staff were retrieved by anonymized, self-declared data or anonymized address-distance calculations.

Similarly, emission factors for medications, manufacturing or construction of buildings, medical equipment and furniture, and transport of materials were not available, which is why these items were excluded from our analyses.

Variables to be provided at/before an annual cut-off date by the centers and relevant to the emission categories mentioned above are given in table 2.

For the individuals filling out the database, IT support was provided by offering the opportunity to call the website operators during working hours. Centers had a choice to provide their data directly via the webform or indirectly, bundled for the years to be submitted to analyses, via CSV files which were transmitted to the website operators and their co-workers for further handling.

**References**

1. https://zenkit.com/en/projects/features/ [Internet]. [cited 2024 Apr 20] Available from: https://zenkit.com/en/projects/features/

2. ecoinvent - Data with purpose. [Internet]. ecoinvent [cited 2024 Mar 9] Available from: https://ecoinvent.org/

3. GEMIS [Internet]. IINAS [cited 2024 Mar 9] Available from: https://iinas.org/en/work/gemis/

4. PROBAS [Internet]. [cited 2024 Jun 21] Available from: https://www.probas.umweltbundesamt.de/datenbank/#/

5. LiveLCA. Products | LiveLCA [Internet]. [cited 2024 Jun 21] Available from: https://livelca.com/products/

6. Gröger J. Digitaler CO2-Fußabdruck.

7. Réalisation d’un bilan des émissions de gaz à effet de serre : secteurs établissements sanitaires et médico-sociaux [Internet]. La librairie ADEME [cited 2024 Mar 9] Available from: https://librairie.ademe.fr/changement-climatique-et-energie/764-realisation-d-un-bilan-des-emissions-de-gaz-a-effet-de-serre-secteurs-etablissements-sanitaires-et-medico-sociaux.html

8. Portail open data de l’ADEME [Internet]. 2024; [cited 2024 Apr 20] Available from: https://data.ademe.fr

9. Health and Wellbeing [Internet]. World Business Council for Sustainable Development (WBCSD) [cited 2024 Mar 9] Available from: https://www.wbcsd.org/Pathways/Health-and-Wellbeing

10. Götz M. Einführung Klimamanagement - Schritt für Schritt zu einem effektiven Klimamanagement in Unternehmen.

11. Anquetin T, Coqueret G, Tavin B, Welgryn L. Scopes of carbon emissions and their impact on green portfolios. Economic Modelling 2022; 115: 105951.

12. Sehgal AR, Slutzman JE, Huml AM. Sources of Variation in the Carbon Footprint of Hemodialysis Treatment. Journal of the American Society of Nephrology 2022; : ASN.2022010086.
